# Incidence of loss to follow-up and associated factors among mothers on antiretroviral therapy in the context of elimination of mother-to-child transmission of HIV in two health districts of Bobo-Dioulasso in Burkina Faso

**DOI:** 10.1186/s12905-025-03658-7

**Published:** 2025-03-29

**Authors:** Béninwendé Léticia Delphine Sakana, Ziemlé Clément Meda, Bernard Ilboudo, Abdoulaye Ouattara, Blahima Konate, Bernard Eric Dabone, Manitu Serge Mayaka, Gueswendé Léon Savadogo, Philippe Van De Perre

**Affiliations:** 1 Centre Muraz, Institut National de Santé Publique, Bobo-Dioulasso, Burkina Faso; 2 Institut Supérieur des Sciences de la Santé, Université Nazi Boni, Bobo-Dioulasso, Burkina Faso; 3 Centre Hospitalier Universitaire Sourô Sanou, Bobo-Dioulasso, Burkina Faso; 4 Institut National des Sciences Sociales, CNRST, DRO, Bobo-Dioulasso, Burkina Faso; 5 Ecole de santé publique de Kinshasa/Université de Kinshasa, Kinshasa, Congo; 6 Département Santé, Université Senghor à Alexandrie, Alexandria, Egypt; 7 Pathogenesis and Control of Chronic and Emerging Infections, University of Montpellier, INSERM, Montpellier, France

**Keywords:** HIV, PMTCT, Adherence, Lost to follow-up, ARV, Mother-to-child transmission

## Abstract

**Introduction:**

Since 2017, Burkina Faso has rolled out a plan to eliminate vertical transmission of HIV (eMTCT). Elimination will only be achieved when mothers have regular follow-up and good compliance with antiretroviral therapy (ART). The aim of this article is to study the incidence and associated factors for loss to follow-up (LTFU ), in women living with HIV (WLHIV) monitored as part of the eMTCT.

**Methods:**

This study consisted of a retrospective analysis of routinely collected data from pregnant women who were screened and initiated ART in the Do and Dafra health districts of Bobo-Dioulasso between 2017 and 2020 as part of the eMTCT. The study ran from May 2023 to March 2024. The Kaplan-Meier model was used to estimate the rate of LTFU; the Cox model was used for identification of factors associated with LTFU and verification of Kaplan-Meier results.

**Results:**

Three hundred and four newly diagnosed pregnant WLHIV were included in this study. The median age of the mothers was 30 [24; 36] years. The incidence of LTFU was 22.9 per 100 person-years. Younger age (HR = 2.40; 95%CI: 1.37–4.20) was associated with a higher incidence of LTFU, whereas urban residence (HR = 0.57; 95%CI: 0.39–0.85), in contrast to rural residence, was associated with a lower incidence of LTFU.

**Conclusion:**

The study showed a high incidence of LTFU, associated with younger age and rural residence, in the eMTCT programme in Burkina Faso.

**Supplementary Information:**

The online version contains supplementary material available at 10.1186/s12905-025-03658-7.

## Background

Vertical transmission of the human immunodeficiency virus (HIV) remains a public health problem, according to the World Health Organisation (WHO) [1]. Worldwide, 130,000 children aged 0–14 newly acquired HIV in 2022, according to the UNAIDS report [2].

Numerous efforts are being made at international level to eliminate vertical transmission (eMTCT) of HIV infection. Since 2013, the WHO has recommended that all HIV-infected pregnant or breast-feeding people should initiate antiretroviral therapy (ART), and that newborns should receive postnatal antiretroviral (ARV) prophylaxis (PNP) from birth until 6 weeks of age in order to reduce vertical transmission of HIV [3, 4]. Numerous studies have demonstrated the benefits of maternal ART. This has contributed to a reduction in the rate of transmission between mother and child [5, 6]. To achieve eMTCT, mothers must be compliant with their treatment, as non-compliance with unsuppressed viral load is associated with failure to protect the child from transmission [7] non-compliance is also responsible for selection of viral mutants resistant to maternal ARV drugs [8, 9]. Regular long-term monitoring of maternal ART is an important part of HIV management and therefore of protecting children, through improved compliance with treatment. One study estimated that about 80% of HIV-infected mothers not receiving ART excrete HIV into breast milk during breastfeeding [10]. This breast milk shedding represents a risk of transmission for the child. Patients who are lost to follow-up (LTFU) during treatment compromise their health, their child’s health and the long-term success of ART programmes [11, 12, 13].

Since 2017, Burkina Faso has rolled out a plan to eliminate vertical transmission of HIV [14]. This plan aims to enable the country to achieve a MCT rate of less than 5% at 12 months for breastfed children [3, 14, 15, 16]. In Burkina Faso, care for women and their children is organised as follows: (i) screening of the mother is carried out during antenatal care at health and social promotion centres, (ii) positive women are registered in the active files of district hospitals where ARVs are provided. They are monitored during pregnancy according to the antenatal care schedule. At delivery, the child is put on nevirapine prophylaxis. The mother continues her ARV treatment for her health and to protect the child during the breastfeeding period. Breastfeeding should be safe for up to 24 months, with the introduction of food supplements from 6 months of age [16].The eMTCT will only be achieved when mothers are regularly monitored and adhere to ART. Retention and good adherence by patients initiating ART remain the cornerstone of long-term viral load suppression [17]. According to UNAIDS, a breakdown of the causes of new paediatric infections showed that interrupted treatment was responsible for the infection of around 10,000 children during pregnancy and 17,000 during breastfeeding in the world [18]. The LTFU throughout the HIV eMTCT cascade remains one of the major threats to the program’s success [19]. It is defined as the absence of renewal of ARV drugs for 3 months or more from the last renewal visit in a mother who has not yet been categorised as deceased or transferred [20, 21]. According to routine data in Burkina Faso, the MTCT rate of HIV was 6.4% for whole the region, and 9.3% in the Hauts Bassins region in 2022 [22]. These rates are above the targets for elimination. No published studies have been conducted to examine the regularity of follow-up under the Burkina Faso program. The program’s success depends on an understanding of the loss of follow-up of mothers and the associated factors to propose strategies for improvement. The present study aimed to analyse the incidence of LTFU and associated factors, in WLHIV followed up as part of the eMTCT.

## Methods

### Study design and setting

The study was carried out in Do and Dafra health districts, in the Hauts Bassins Region of Burkina Faso.

The two health districts (Do and Dafra) provide care for the population of the city of Bobo-Dioulasso; they are part of the 7 districts in the Haut Bassins region (Do Dafra, Dandé, Houndé, Lena, Karangasso vigué orodara). They represent the first level of the health pyramid in Burkina Faso (see supplement 1). These two districts are made up of 52 peripheral public health facilities (including 29 in rural areas and 23 in urban areas), 150 private health facilities, and two district hospitals. The two district hospitals are following people living with HIV monitored since 2010, cumulating 3.072 patients (1.822 in Do and 1.250 in Dafra) as of march 2024.

### Type and period of study

This study consists of a retrospective analysis on data routinely collected from pregnant women screened and initiated ART as part of the HIV eMTCT program from 2017 to 2020. The study ran from May 2023 to March 2024.

### Study population

The study population was WLHIV newly screened and diagnosed with HIV as part of eMTCT and initiated on ART in the Do and Dafra health districts from 2017 2020.

They were not declared transferred or deceased, and with the inclusion date filled in the districts log books and medical records.

### Sampling and sample size

**Fig. 1:**
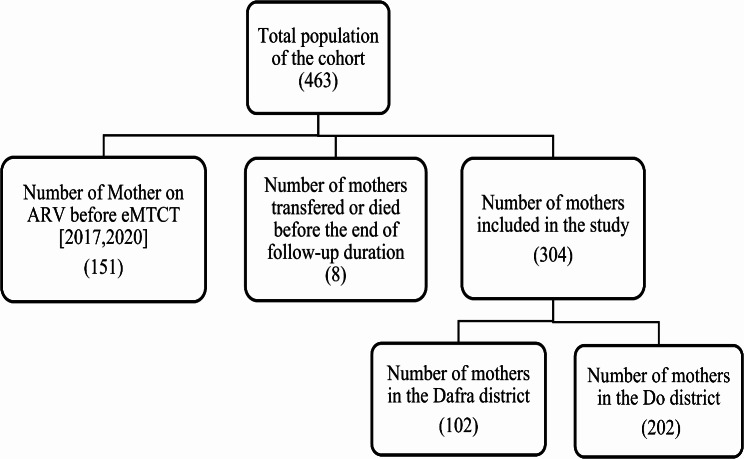
Flow chart of study participants

An exhaustive sample of women followed up in the Do active file in Dafra and meeting the inclusion criteria was taken. A total of 463 women were monitored as part of the eMTCT between 2017 and 2020, including 342 in Do and 121 in Dafra (Fig. 1).

### Study variables

The independent variables were type of HIV (HIV1, HIV2, HIV1&2) and socio-demographic variables: level of education (none, primary, secondary, tertiary), age (adolescents and young people aged 15 to 24, young adults aged 25 to 34 and adults aged 35 or over), residence (rural or urban), mother’s occupation (employed, self-employed), number of children (no child, one child, two or more children) and marital status (couple, non-couple).

The dependent variable of the study was LTFU in the context of HIV eMTCT. A woman was considered lost to follow-up if she had missed her ART renewal for 3 months or more from the last renewal visit and had not yet been categorised as deceased or transferred, from the date of inclusion to 18 months after delivery. The duration of follow up of each woman is showed in supplement 2.

### Data sources

The data were obtained from the ESOPE software and the pharmacy dispensing file of the two districts in Do and Dafra. ESOPE is a clinical monitoring tool for patients on ART used routinely in Burkina Faso. Clinical data were linked to dispensing data using each patient’s unique identification number.

### Statistical analysis

The data was extracted and exported in Excel format. The analysis was carried out using R (version 4.3.1). Descriptive statistics were used to determine the distribution of the socio-demographic characteristics of the study population. The results were presented as proportions.

Univariable Cox analysis was used to identify factors associated with loss to follow-up. Variables showing a significant association at the 30% threshold were selected for the construction of a multivariable Cox model. The Schoenfeld test was used to assess the risk-proportionality assumption of the Cox model. In parallel, the Kaplan-Meier curve and the log Rank and Gehan-Breslow tests were used to compare the incidence of LTFU between the different modalities of the associated independent variables. The minimum accepted significance level for the statistical tests was 5%.

## Results

### Study participants

A total of 304 women were included in the study. The majority of these women were in the Do district (66.45%). (Fig. 1).

### Socio-demographic characteristics of study participants

The median age of the participants was 30 years [range: 24 to 36 years]. The majority of women lived in urban areas, were not in paid employment or education, lived with a partner, had fewer than two children, and were HIV 1-infected (Table 1).

**Table 1:** Socio-demographic characteristics of study participants

Variable	*n* (%)
**Age category**	
≥ 35	116 (39.6)
25–34 years	122 (41.6)
15–24 years	55 (18.8)
**Level of education**	
None	149 (54.8)
Primary	52 (19.1)
Secondary and above	71 (26.1)
**Profession**	
Self-employment	269 (94.1)
Salaried employment	17 (5.9)
**Residence**	
Rural	93 (31.8)
Urban	199 (68.1)
**Number of children**	
No child	80 (28.88)
1 child	69 (24.91)
≥ 2 children	128 (46.21)
**Marital status**	
Single	24 (8.79)
Cohabiting	9 (3.30)
Divorced	6 (2.20)
Maried	224 (82.05)
Windowed	10 (3.66)
**Type of HIV**	
HIV1	275 (96.5)
HIV1&2	6 (2.1)
HIV2	4 (1.4)
**District**	
Dafra	102 (33.55)
Do	202 (66.45)

### Results of loss to follow-up

The follow-up time for the 304 women was 444 person-years. The incidence of loss to follow-up was 22.9 per 100 person-years. 102/304 women were lost to follow-up during the study period, representing 33.55% of the sample. The Do health district had the highest number of women lost to follow-up (97).

### Factors associated with LTFU

**Fig. 2:**
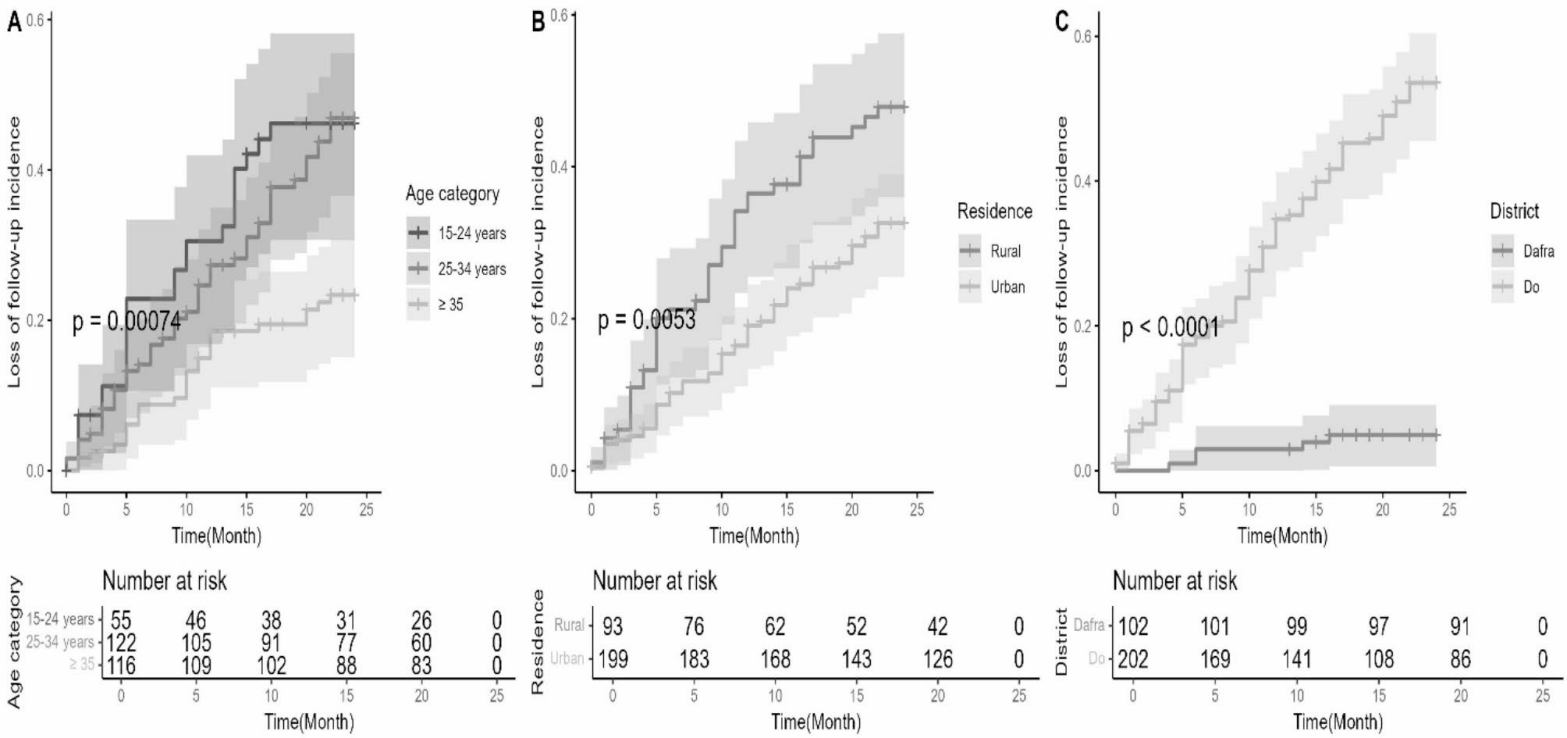
Evolution in LTFU with treatment as a function of time (Kaplan-Meier curves) by age group (**A**) by residence (**B**) and District (**C**)

The log-rank test results showed a significant association with age (Chi2 = 7.8; df = 1; *p* = 0.005), residence (Chi2 = 14.4; df = 2; *p* < 0.001) and district (Chi2 = 60.4; df = 1; *p* < 0.001), and the Kaplan Meier curves (Fig. 2)further illustrate these associations. At month five, 20% of women in rural areas were lost follow-up, versus at 14 months in urban women. 20% of women aged 15 to 24 were lost follow-up at 5 months. This situation was observed at 9 months for women aged 25 to 34 and 20 months for whose aged 35 and over. 20% of women losing follow-up at 7 months for the district of Do.

The Cox analysis confirmed Kaplan Meier results, only young age residence in a rural area and the following in Do health district were associated with a higher incidence of LTFU in women undergoing eMTCT (Table 2).

**Table 2:** Univariable and multivariate analysis (Cox model) of factors associated with LTFU in women undergoing treatment as part of the eMTCT strategy (*n* = 292)

Variables	Non ajusted				Ajusted			
	HR^1^		95% CI^1^	*p*-value	HR^1^		95% CI^1^	*p*-value
**Age category**				**< 0**,**001**				**0**,**001**
≥ 35	—		—		—		—	
25–34 years	2.24		1.40, 3.59		1.93		1.20, 3.09	
15–24 years	2.45		1.41, 4.27		2.40		1.37, 4.20	
**Level of education**				0.61				
None	—		—					
Primary	0.78		0.4 ;1.4					
Secondary and above	0.84		0.5;1.4					
**Profession**				0.89				
Non-salaried employee	—		—					
Salaried employee	0.94		0.4;2.2					
**Résidence**				**0.007**				
Rural	—		—					
Urban	0.57		0.39, 0.85					
**Number of child**				0.43				
No child	—		—					
1 child	0.79		0.45, 1.37					
≥ 2 children	0.75		0.46, 1.23					
**Marital status**				0.051				
Not in couple	—		—					
In couple	2.01		0.9;4.3					
**District**				**< 0.001**				**< 0.001**
Dafra	—		—		—		—	
Do	14.8		6.00, 36.3		13.2		5.36, 32.5	
**Type of HIV**				0.57				
HIV1	—		—					
HIV1&2	1.93		0.61;6.1					
HIV2	0.74		0.10;5.3					
^1^HR = Hazard Ratio, CI = Confidence Interval								
		**Non ajusted**				**Ajusted**		

## Discussion

This study, carried out in two health districts in the city of Bobo-Dioulasso, Burkina Faso showed a high incidence of LTFU of 22.9 per 100 person-years for women monitored as part of the eMTCT. More than a third of the mothers followed up were declared lost to follow-up. Age and place of residence were associated with loss to follow-up. Occupation, marital status, level of education, number of children and type of HIV were not associated with LTFU.

Our study is the first to examine the incidence of LTPU in the context of eMTCT in Burkina Faso. It could enable the country to implement strategies to improve the eMTCT programme.

The high incidence of LTFU shows that women are not adhering to the strategy. The new WHO recommendations in 2017, propose starting treatment for any patient screened, even in the absence of a paraclinical work-up; this should support a low incidence rate [23]. This strategy avoids patients dropping out before inclusion, due to a lack of financial resources to carry out biological tests [23]. The high incidence of LTFU can explain the rate of HIV MTCT, which is 9.3% according to routine data from the Hauts Bassin region [22].

Young age was strongly associated with a higher incidence of loss to follow-up Fear of stigmatisation lead to non-adherence to treatment [24, 25] and may explain why patients lose their follow-up. Our findings are similar to those of Mushy and colleagues in Tanzania [24], reporting a high risk of LTFU in young people aged 15–24.

Place of residence was associated with loss of attendance. Women living in rural areas were less likely to attend than those living in urban areas. Since 2014, Burkina Faso has allowed HIV patients to be treated at health and social promotion centres [26]. Managers of peripheral health facilities with HIV-infected patients deliver ARVs at the district level, and patients obtain supplies directly from the peripheral centre. This should improve adherence because patients are no longer obliged to travel to district hospitals. Our results show that despite this, the problem of follow-up persists in rural areas. In Burkina Faso’s national guidelines, health workers are accompanied by psychosocial counsellors (mediators) in carrying out eMTCT. They carry out counselling activities for screening, compliance consultations and searches for people who have been lost to follow-up. This is not done in rural areas, and may be linked to the high incidence of people dropping out of follow-up in rural areas [16]. Our results are similar to those of studies conducted in Burkina Faso and Ethiopia, which found that living in a rural area was associated with LTFU [27, 28]. More than a third of women missed their supply appointment over a period of three successive months. A study comparing women who discovered their status during pregnancy with those who knew they were infected and who joined the eMTCT programme during pregnancy showed that newly diagnosed pregnant women were less compliant with treatment than those who knew they were infected before the current pregnancy [29, 30]. This could explain the rate of loss to follow-up, as we only took newly diagnosed women. They drop out of follow-up at the end of the strategy when the child is protected, and no longer follow up for their own health. Adherence consultations need to be stepped up in the context of eMTCT to give mothers a better understanding of the disease and the drawbacks associated with non-adherence and loss of adherence to treatment (resistance, transmission).

The incidence of LTFU in this study was higher than the 9.4 per 1000 person-months of Azanaw et al. [31]. Azanaw and al carried out a retrospective follow-up study conducted among pregnant and lactating women with ART (option B+) at health facilities in Northwest Ethiopia. Also, Geremew et al. [30] reported a 12.04 per 1000 person-months incidence among women who were enrolled for option B + prevention of mother-to-child transmission service between June 2013 and March 2021 in Pawi district health facility. The high rate in our study could be explained by the fact that we followed each of the women for the entire duration of the eMTCT (until the child is 18 months old), which is not the case in the study by Geremew and colleagues. This study included all women whose first appointment took place at least one month before the data extraction date. The study by Azanaw et al. only included pregnant and breastfeeding women who had been enrolled in services for the prevention of vertical transmission of HIV for at least three months before the end of data collection. These studies include also women who were on ART before their inclusion in PMTCT.

### Limits

Our study was based on retrospective data collection from two merged routine databases (clinical and dispensing data). It has limitations linked to the absence of certain information on clinical (e.g. WHO stage) and paraclinical (e.g. viral load testing) and sociodemographic data. Our analysis of LTFU was limited to the supply of drugs in pharmacies. Another limitation of our study is the lack of traceability in the follow-up of patients on ART. Some mothers considered as LTFU could have died or transferred themselves, which could have increased our rate of LTFU. We only collected data from women newly diagnosed during the eMTCT pregnancy. Data from women who were already known to be positive before their pregnancy and who were being monitored as part of the eMTCT were not taken into account, which prevents us from generalising the results to all women being monitored as part of the eMTCT. However, our findings could justify improved follow up strategies in general, and in particular in mothers in rural areas.

## Conclusion

In conclusion, our study shows a high incidence of LTFU. It shows an association between the place of residence, age, and the timing of LTFU. Strategies need to be found for better follow-up of mothers during and after eMTCT. Follow-up must also be stepped up for women in rural areas. In-depth studies on compliance (daily intake of medication) must be carried out, and strategies to retain the mother-child couple in the program should be found to achieve the objectives of eMTCT.

## Electronic supplementary material

Below is the link to the electronic supplementary material.

Supplementary Material 1

Supplementary Material 2

## Data Availability

The data used in this study are not publicly available. They can be obtained from those involved in the care of people living with HIV in the two district hospitals.

## Abbreviations

ART: Antiretroviral Treatment
ARV: Anti‑retroviral
eMTCT: Elimination of mother-to-child transmission
HIV: Human Immunodeficiency Virus
LTFU: Loss to follow-up
MTCT: Mother‑to‑child transmission
UNAIDS: United Nations Programme on HIV/AIDS
WHO: World Health Organisation
WLHIV: Women Living with HIV
