# Supplementary figures and images for: Incidence of loss to follow-up and associated factors among mothers on antiretroviral therapy in the context of elimination of mother-to-child transmission of HIV in two health districts of Bobo-Dioulasso in Burkina Faso
Source: BMC Womens Health. 2025 Mar 29;25:148. doi: 10.1186/s12905-025-03658-7 (PMC11954237; doi:10.1186/s12905-025-03658-7)

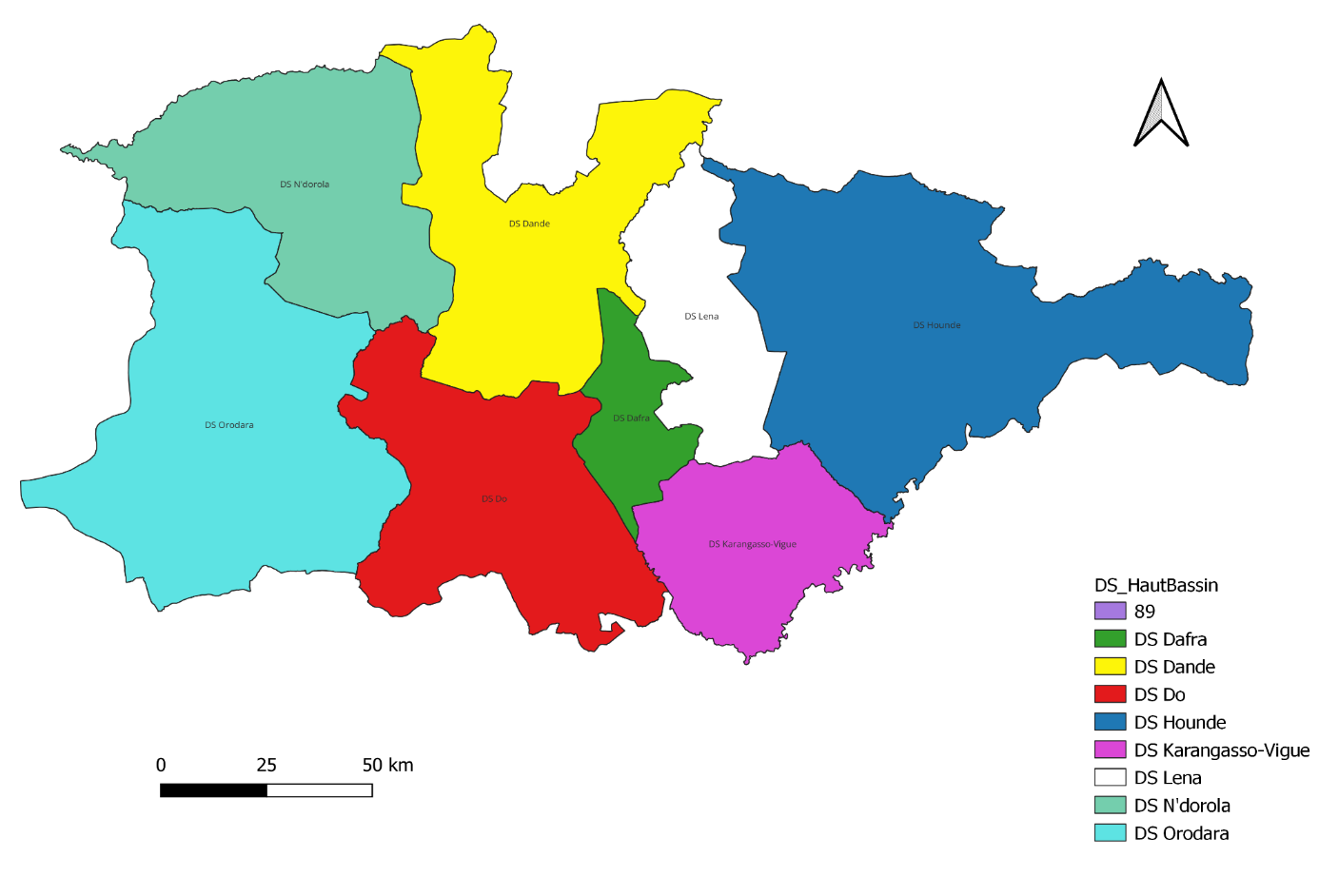


Supplement 1 : Health map of the Hauts Bassins region

Supplement: Supplementary file 1 — Supplementary Material 1 [file 12905_2025_3658_MOESM1_ESM.docx]

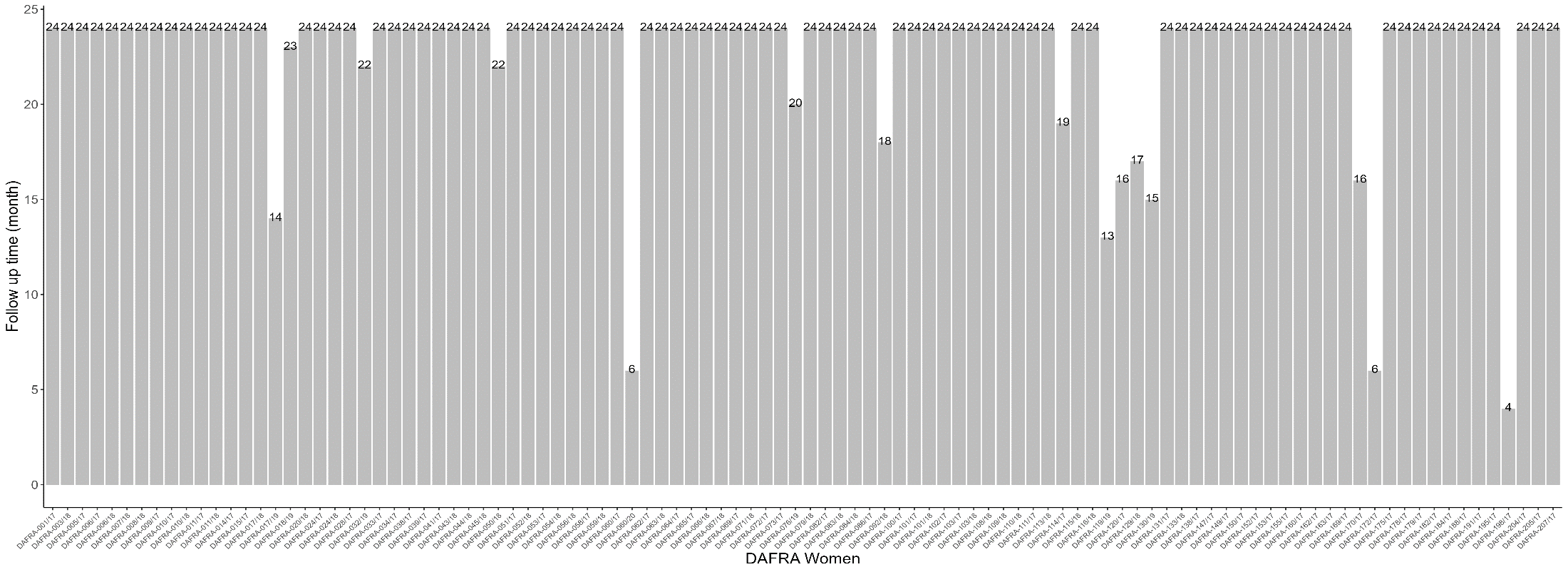

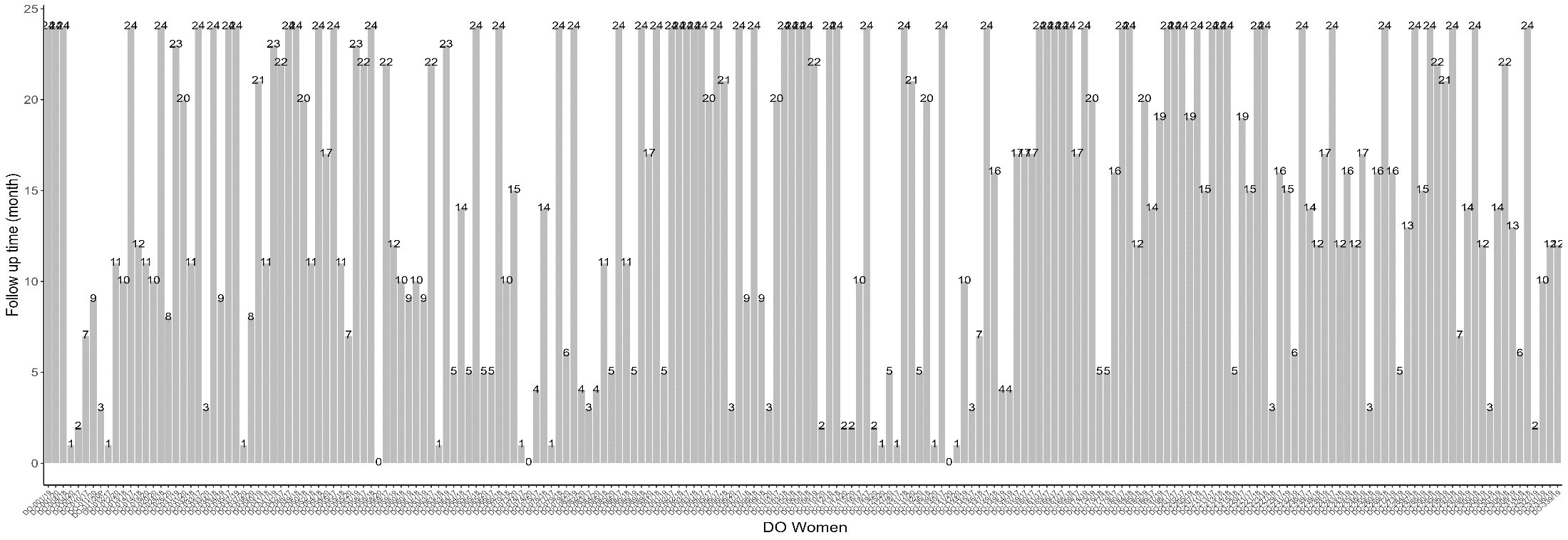


Supplement 2: duration time of follow up for each participant in the two districts

Supplement: Supplementary file 2 — Supplementary Material 2 [file 12905_2025_3658_MOESM2_ESM.docx]
